# Trends in 1-Month Breastfeeding Before, During, and After the Coronavirus Disease 2019 Pandemic: A Retrospective Single-Center Study in Japan

**DOI:** 10.3390/children13081105

**Published:** 2026-08-18

**Authors:** Kyosuke Tabata, Nao Umebayashi, Airi Tanaka, Emiko Suzuki, Yayoi Murano, Tomoyuki Nakazawa, Ken Sakamaki, Daisuke Yoneoka, Hiromichi Shoji

**Affiliations:** 1 Division of Pediatrics, Tokyo Metropolitan Toshima Hospital, Tokyo 173-0015, Japan; 2 Department of Pediatrics, Faculty of Medicine, Juntendo University, Tokyo 113-0033, Japan; 3 Division of Obstetrics and Genecology, Tokyo Metropolitan Toshima Hospital, Tokyo 173-0015, Japan; 4 Division of Epidemiology, National Institute of Infectious Diseases, Japan Institute for Health Security, Tokyo 162-8655, Japan; 5 Graduate School of Medicine, The University of Tokyo, Tokyo 113-0033, Japan

**Keywords:** COVID-19, pandemic, breastfeeding

## Abstract

**Highlights:**

**What are the main findings?**

• Breastfeeding rate declined during and after COVID-19 pandemic.

**What are the implications of the main findings?**

• Disruptions in educational opportunities, reduced social interactions, and changes in health-seeking behaviors adversely affected breastfeeding practices.

**Abstract:**

**Background/Objectives**: Breastfeeding confers important health benefits for mothers and children. The coronavirus disease 2019 (COVID-19) pandemic disrupted childcare practices and maternal behaviors, with some effects persisting beyond the acute phase. This study aimed to evaluate the impact of the COVID-19 pandemic on breastfeeding. **Methods**: This retrospective single-center study included full-term infants born at Tokyo Metropolitan Toshima Hospital between January 2019 and January 2024. Data was routinely collected. The participants were categorized into those born before, during, and after the pandemic. The rates of exclusive breastfeeding and volume of formula milk at 1-month health checkups were compared across the groups using the chi-square test and regression analysis. **Results**: A total of 812 (30.1%), 1602 (61.0%), and 214 (8.1%) infants born before, during, and after the pandemic, respectively, were analyzed. The rates of exclusive breastfeeding differed significantly among the groups, (42.2%, 28.1%, and 22.9%, respectively). Concurrently, formula milk consumption increased during the study period, with significant differences observed between those born before and during the pandemic and between those born before and after the pandemic. **Conclusions:** Breastfeeding rate declined during the COVID-19 pandemic and the effect persisted after the pandemic. We speculate this trend may be attributed to the loss of educational opportunities and reduced interactions among mothers. Another contributing factor was the shift in the sources of information for maternal decision-making. After the pandemic, reliance on social media as a primary source of guidance increased; however, much of this information was less evidence-based and potentially inaccurate. Healthcare providers should recognize these challenges and prepare for future pandemics.

## 1. Introduction

Breastfeeding benefits that provide substantial short- and long-term health gains for both infants and mothers are well established [1], and breastfeeding’s promotion remains a critical public health priority for child health all over the world. In recent years, several studies have reaffirmed the protective effects of breastfeeding on infant immunity, neurodevelopment, and maternal health, emphasizing its importance even in high-income countries. [2,3,4] Taken together, these findings highlight that breastfeeding remains a cornerstone of early-life health and a critical public health priority.

The coronavirus disease 2019 (COVID-19) pandemic introduced unprecedented challenges to maternal and child health. COVID-19 typically causes milder symptoms in children than in adults [5,6], but the pandemic profoundly disrupted daily lives, affecting physical activity, social interaction, and access to healthcare services [7,8,9]. Although breastfeeding in mothers with COVID-19 was controversial at the beginning of pandemic [10,11], the World Health Organization (WHO) recommended breastfeeding [12].

In the current global era, infectious diseases spread more readily because of increased mobility and urbanization, and experts widely anticipate that another pandemic will occur in the foreseeable future [13]. Understanding how breastfeeding practices changed during COVID-19 is therefore essential not only for evaluating the pandemic’s impact but also for strengthening preparedness for future public health emergencies. However, the extent to which the COVID-19 pandemic influenced breastfeeding practices remains insufficiently understood.

Given these considerations and emerging evidence from several regions that show the pandemic affected breastfeeding in multiple ways, including changes in hospital policies, maternal mental health, and access to professional and peer support [14,15], the present study focused on breastfeeding practices among infants in a metropolitan hospital setting and aimed to assess how the COVID-19 pandemic affected exclusive breastfeeding rates and formula milk consumption. By examining data across pre-pandemic, pandemic, and post-pandemic periods, this study seeks to contribute updated evidence that can inform future maternal–child health strategies and guide preparedness for subsequent pandemics.

## 2. Materials and Methods

This study is an observational retrospective single-center study following STROBE guidelines. The study participants were infants delivered at Tokyo Metropolitan Toshima Hospital between January 2019 and January 2024 who underwent a routine 1-month health checkup. The following information was extracted from medical records: feeding methods (breastfeeding status and volume of formula milk), date of delivery, method of delivery (vaginal or cesarean section), sex, gestational age, and birth weight. Feeding strategies were defined based on the status at the 1-month health checkup. Exclusive breastfeeding was defined as feeding with breast milk only at that time point. Mixed feeding referred to infants receiving both breast milk and formula milk, and formula feeding referred to infants fed exclusively with formula milk. Participants in the present study were term infants without complications, and exclusion criteria included infants born preterm, those hospitalized at the one-month health checkup, those from multiple pregnancies, and those with congenital diseases. We excluded infants who experienced preterm birth, hospitalization at the time of the 1-month health checkup, multiple pregnancy, and congenital diseases because these conditions are well-known to influence breastfeeding outcomes independently of the pandemic.

At Tokyo Metropolitan Toshima Hospital, parents attend an educational class between 22 and 30 weeks of gestation, approximately 2–4 months before delivery. This education program provides information on breastfeeding, newborn care, and postpartum recovery, and has an impact on feeding decision-making. The first patient with COVID-19 was reported on January 15, 2020, and the disease was classified as a class 5 infectious disease in Japan at the end of the pandemic. Therefore, participants were categorized into three groups based on their date of delivery: before the pandemic (between January 2019 and March 2020), during the pandemic (April 2020 to September 2023), and after the pandemic (October 2023 to January 2024). During the pandemic, isolation policies were determined individually by each hospital, and no nationwide policy was mandated by the government. At our hospital, educational classes were suspended, partner attendance at delivery was prohibited, SARS-CoV-2 PCR testing was required prior to admission, and mask-wearing was mandatory within the facility.

Participant characteristics were analyzed using analysis of variance for continuous variables and the chi-square test for categorical variables. Feeding status was evaluated using two approaches. First, the proportion of infants who were exclusively breastfed was compared between subgroups using Fisher’s exact test. Then multivariate logistic regression analyzing exclusive breastfeeding (yes/no) was performed, with factors associated with exclusive breastfeeding including delivery mode (normal delivery vs. cesarian section) and maternal age [16]. We also assessed the association between the study period and exclusive breastfeeding as an independent factor. Second, the amount of formula milk consumed was compared among the three groups using one-way analysis of variance (ANOVA). When significant differences were detected, pairwise comparisons were conducted using *t*-tests with Holm’s method to adjust for multiple testing. All statistical analyses were performed using R version 4.1.1 (R Foundation for Statistical Computing, Vienna, Austria).

This study was approved by the Ethics Committee of Toshima Hospital (approval number RinJin6-22). All data were extracted in a fully anonymized, non-identifiable format, and no personal information was included at any stage of the analysis. The study was approved by the Institutional Ethics Committee of Tokyo Metropolitan Toshima Hospital. The requirement of obtaining informed consent was waived as the study involved minimal risk and used de-identified data. In accordance with the institutional guidelines, an opt-out option was provided through public disclosure, allowing patients to decline the use of their data.

No generative artificial intelligence was used in the preparation of this manuscript.

## 3. Results

### 3.1. Study Participants

#### 3.1.1. Number of Participants

**Figure 1:**
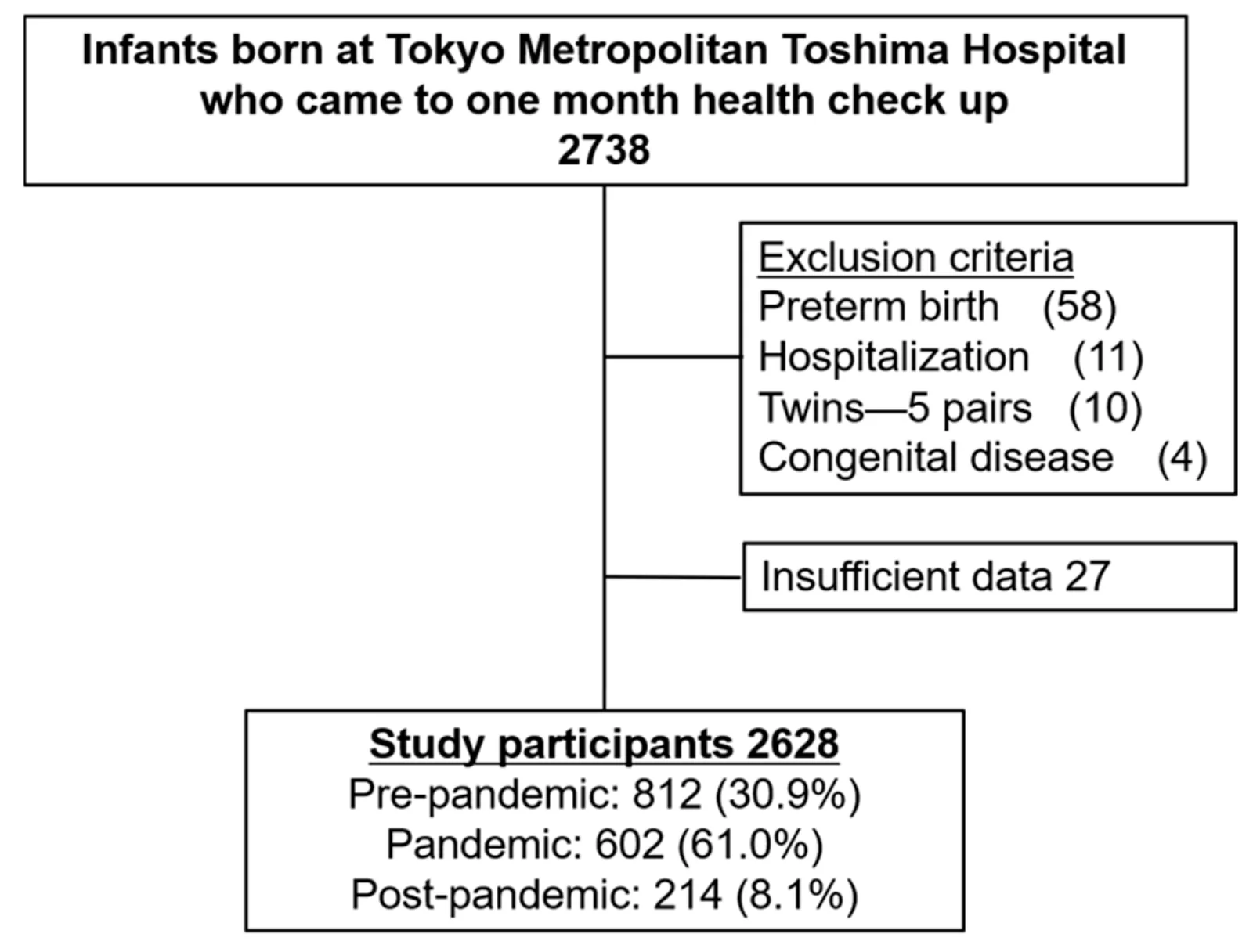
Flow diagram of participant selection.

The participant selection process is illustrated in Figure 1. A total of 2738 infants were born at Tokyo Metropolitan Toshima Hospital and underwent a 1-month health checkup during the study period. Infants with factors associated with poor breastfeeding outcomes—including preterm birth, hospitalization at the time of the 1-month health checkup, multiple pregnancies, and congenital diseases—were excluded. In addition, 27 infants with insufficient data were excluded. After applying the exclusion criteria, 2628 infants were included in the final analysis. Of these, 812 infants (30.9%) were classified in the pre-pandemic group, 1602 (61.0%) in the pandemic group, and 214 (8.1%) in the post-pandemic group.

#### 3.1.2. Participant Characteristics

The participants’ characteristics are presented in Table 1. Gestational age, sex, delivery method, and birth weight did not significantly differ among the groups.

### 3.2. Breastfeeding Practice

#### 3.2.1. Distribution of Feeding Methods

At the 1-month health checkups, the proportions of infants receiving exclusive breastfeeding, mixed feeding (breast milk and formula), and formula feeding alone differed significantly among the groups. The proportion of exclusive breastfeeding significantly decreased during the study period. The proportion of infants receiving exclusive breastfeeding differed significantly between those born before and during the pandemic (42.2% vs. 28.1%, *p* < 0.01) and between those born before and after the pandemic (42.2% vs. 22.9%, *p* < 0.01) (Table 2).

#### 3.2.2. Exclusive Breastfeeding

Logistic regression analyzing exclusive breastfeeding showed significantly decreased odds ratios during and after the pandemic. Other factors including delivery mode and maternal age had significant odds ratios (Table 3).

#### 3.2.3. Amount of Formula Milk in Infants Receiving Mixed Feeding

**Figure 2:**
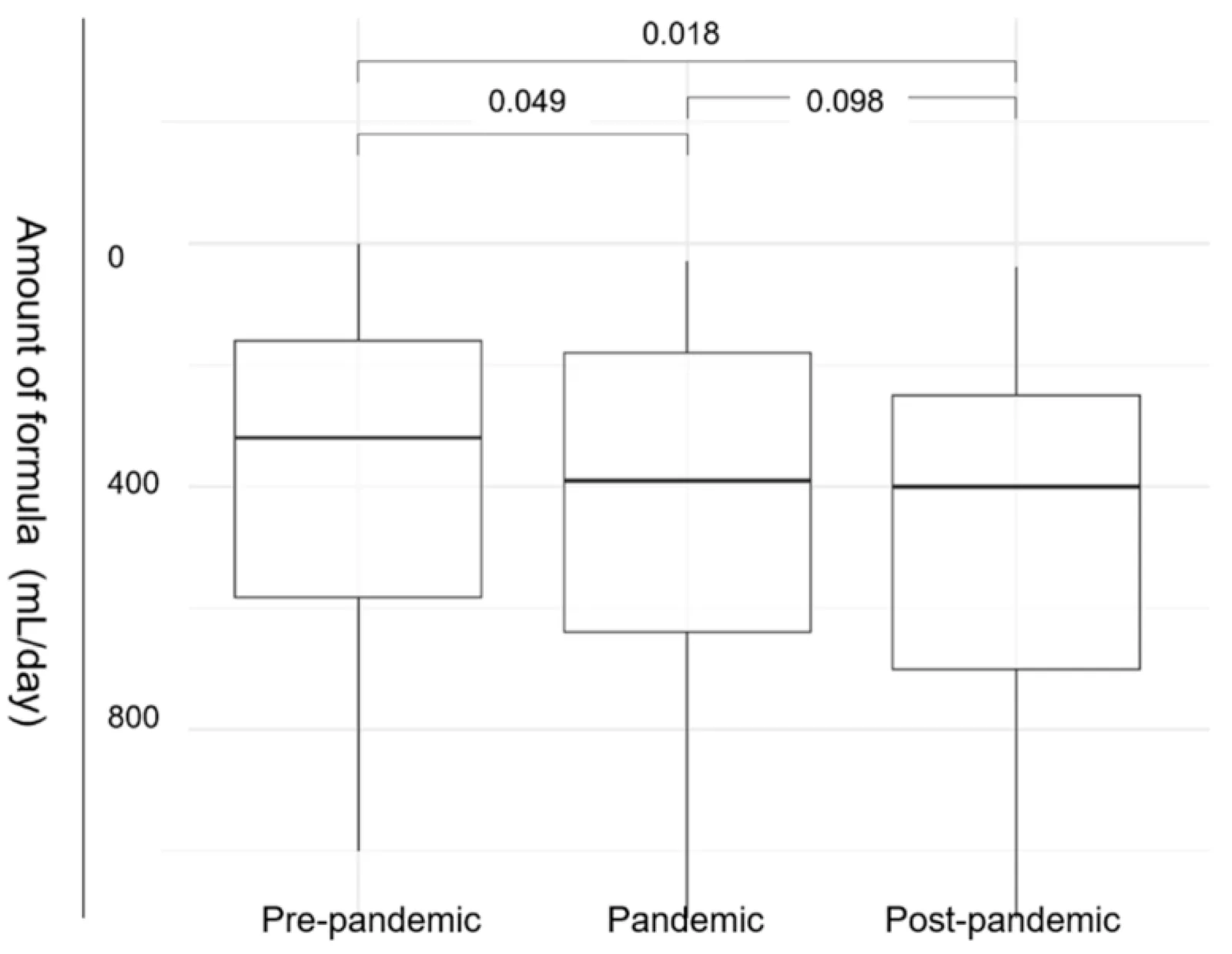
Formula milk volume among infants receiving mixed feeding: increased amount of formula milk indicates decreased mother’s milk amount.

Figure 2 presents a boxplot of formula milk volume, stratified by subgroup. The mean daily formula milk intake differed significantly between the before- and during-pandemic groups (378.3 mL/day vs. 411.9 mL/day, *p* = 0.049) and between the before- and after-pandemic groups (378.3 mL/day vs. 455.5 mL/day, *p* = 0.018).

## 4. Discussion

This study examined changes in breastfeeding practices before, during, and after the COVID-19 pandemic. Across the study period, exclusive breastfeeding rates declined markedly, with significant reductions observed during the pandemic and further decreases in the post-pandemic period. Logistic regression analysis confirmed that being born during or after the pandemic was independently associated with lower odds of exclusive breastfeeding, even after adjusting for maternal age and delivery mode. In addition, among infants receiving both mother’s milk and formula milk, the volume of formula milk increased progressively from the pre-pandemic to the post-pandemic period, suggesting a shift toward greater reliance on formula supplementation.

A large cross-sectional survey conducted in Europe reported a decline in exclusive breastfeeding rates during the COVID-19 pandemic [14]. Moreover, despite recommendations to continue breastfeeding even among mothers infected with SARS-CoV-2, several studies have shown decreased breastfeeding rates among mothers with COVID-19 [17]. A study from Japan also identified the COVID-19 pandemic as a factor associated with reduced exclusive breastfeeding [18]. Even in a baby-friendly hospital that promotes breastfeeding, a decline in breastfeeding rate was reported [19]. Reasons for this decrease in breastfeeding were indicated as insufficient support and maternal mental health problems due to the COVID-19 pandemic, while decision-making and knowledge about breastfeeding did not change during the pandemic [20]. In contrast, several studies have reported that, during the pandemic, there were either no adverse effects on breastfeeding or that the pandemic had a positive impact on breastfeeding [15,21,22,23]. However, the majority of studies have documented decreases in breastfeeding rates during the pandemic, which is consistent with our findings. One study that did not observe a decline concluded that online support played a beneficial role. Based on these findings, it can be inferred that the type of support available during the pandemic—including social, medical, and peer support—may also influence breastfeeding practices. Although these studies have reported heterogeneous effects of the COVID-19 pandemic on breastfeeding practices, a consistent conclusion that has emerged over time is the importance of maintaining fundamental breastfeeding-supportive care including skin-to-skin contact and other practices that facilitate early mother–infant attachment, even in the context of maternal SARS-CoV-2 infection. And our study added the finding that the influence remained even after the pandemic ended [24,25,26,27].

Although, peer support and education are known to be important factors for breastfeeding, [28,29] parental educational classes were canceled during the COVID-19 pandemic period in many hospitals, including our hospital. Moreover, government-mandated social distancing policies limited in-person communication opportunities for parents to interact with one another, which reduced opportunities for interactions between mothers and family members, other expecting mothers, and healthcare providers. Numerous studies have reported increased social isolation among the elderly population during the pandemic [8]; however, only a few studies have examined its impact in younger populations including pregnant women and new parents, who were also affected [9]. Consequently, parents may have had limited access to accurate information and fewer opportunities to establish supportive relationships with other parents during and after pregnancy. Reduced opportunities for peer support, informal learning, and shared problem-solving may have hindered parents’ ability to obtain accurate breastfeeding information or seek timely assistance when difficulties arose. Such disruptions are particularly important because social support is a well-established determinant of breastfeeding initiation and continuation [30]. In addition to a decrease in peer support, several studies have documented that the COVID-19 pandemic led to substantial disruptions in professional perinatal care: a Cochrane review demonstrated that professional lactation support significantly improves breastfeeding outcomes [29]. A large systematic review reported that perinatal care systems were widely affected, with restrictions on routine support practices that are essential for breastfeeding establishment [31]. Another study highlighted that the reduced access to breastfeeding support during the pandemic contributed to difficulties in maintaining breastfeeding [26]. At our institution, midwives also experienced a loss of opportunities to provide hands-on breastfeeding support during the pandemic. As a result, the temporary reduction in clinical exposure may have led to decreased practical experience among staff, which could have contributed to suboptimal breastfeeding support in the immediate post-pandemic period.

However, one study reports an increase in exclusive breastfeeding during the COVID-19 pandemic, and discusses the effects of changes in work patterns. For example, increased opportunities to work from home and reduced working hours were suggested as potential contributors to higher breastfeeding rates in certain populations [32]. In Japan, similar changes in work style occurred; however, fathers also worked from home, which may have influenced feeding practices in different ways, such as an increase in formula milk consumption, in our study. In addition, some of these previous studies did not clearly describe their recruitment methodology, making it difficult to determine how representative their samples were. In contrast, our study used routinely collected clinical data from a defined population, which may more accurately reflect the actual situation during the pandemic. Additionally, several studies assess the psychological effects of the pandemic on breastfeeding mothers [33,34], which may have also contributed to a decrease in breastfeeding rate.

Another consequence of the COVID-19 pandemic is the shift in sources of health-related information. Social isolation during the pandemic changed how individuals accessed healthcare-associated information, with decision-making increasingly influenced by social media [35,36]. In Japan, posts recommending formula milk feeding have been frequently observed. However, such information is often biased and lacks medical evidence. However, news sources on social media exhibit systematic bias and differing sentiment patterns [37], which may influence how individuals interpret health-related information. Studies have shown that social media can promote unnecessary medical interventions, such as elective cesarean sections [38], and can influence parental decision-making in ways that diverge from clinical recommendations. Younger populations, who are more likely to rely on social media than older adults, may be particularly vulnerable to such misinformation. Healthcare providers working with pregnant women and new parents should therefore be aware of the influence of digital platforms and consider integrating media literacy and evidence-based online resources into perinatal education. However, social media is often an useful tool for successful intervention [39] and holds the potential to be particularly beneficial for this generation.

However, these assessments, including declining access to breastfeeding education, diminished social interaction, and increased reliance on online information, were not able to be measured directly in our study and are discussed as speculation. Therefore, these interpretations should be considered speculative and assessed in future studies.

The sustained decline in exclusive breastfeeding observed in the post-pandemic period suggests that the effects of the pandemic may persist beyond the acute phase of the crisis. This pattern aligns with broader concerns that children and young families—often among the most vulnerable groups [40]—were disproportionately affected during the pandemic and may continue to experience long-term consequences. Previous research has highlighted that children are frequently “left behind” during public health emergencies, with disruptions to healthcare, education, and social support systems contributing to widening inequalities [40,41]. Our findings reinforce the need for targeted interventions to support breastfeeding and early child health during future crises.

While these studies primarily focus on the immediate effects of the pandemic and the disruptions that occurred during the acute phase, it is also important to consider how breastfeeding practices evolved after the pandemic. Our study adds new evidence by demonstrating that the decline in exclusive breastfeeding persisted even after the pandemic formally ended. Few previous studies have examined post-pandemic trends, and our results suggest that the loss of antenatal education, diminished peer interaction, and increased reliance on social media for health information may have had lingering effects on parental feeding decisions. In addition, the disruption of education and training for young healthcare providers during the pandemic may have contributed to suboptimal outcomes in the post-pandemic period. This extended decline underscores the need to consider not only acute pandemic disruptions but also their longer-term consequences for maternal behaviors. Moreover, in our previous research, we demonstrated that evaluating not only whether infants were exclusively breastfed but also the amount of formula milk they consumed—which reflects the volume of breast milk actually consumed—is important. This study incorporated that insight into its design [42].

This study has several limitations. As a single-center study, the findings may not be generalizable to other regions or healthcare settings. Differences in healthcare infrastructure, breastfeeding support systems, and social or cultural contexts may influence how the pandemic affected infant feeding practices, and caution is needed when applying these results to other populations. However, the hospital’s standardized perinatal care procedures remained consistent throughout the study period, except for changes necessitated by infection control policies, which strengthens the internal validity of the results. Another limitation is that the study is an observational study and relied on routinely collected clinical data, which did not capture the underlying reasons for parental feeding decisions. Qualitative studies exploring parental experiences, perceptions of support, and exposure to online information would provide valuable insights into the mechanisms underlying the observed trends. Moreover, several important determinants of breastfeeding—such as parity, maternal occupation, education, socioeconomic status, previous breastfeeding experience, and maternal illness—were not available in our dataset and we could not include these factors in our analysis. These factors are well-established influences on breastfeeding practices, as highlighted in the previous literature [16,43], and should be assessed in future studies. Finally, the post-pandemic period in our study was relatively short, resulting in a smaller sample size compared with the other periods. This limited duration may reduce the precision of estimates for the post-pandemic group. In addition, breastfeeding practices may continue to evolve as social conditions and perinatal care systems stabilize after the pandemic. Therefore, further studies with longer post-pandemic follow-up periods are warranted.

In recent decades, several pandemics and large-scale infectious disease outbreaks have occurred, and experts anticipate that similar events will continue to emerge. [13] The COVID-19 pandemic highlighted the vulnerability of children and young families, who were often overlooked in policy responses despite facing significant disruptions to daily life and healthcare access. Understanding the long-term effects of the pandemic on breastfeeding and early child health is essential for strengthening preparedness for future public health emergencies. Developing resilient support systems, enhancing access to evidence-based information, and ensuring continuity of perinatal care—even during crises—will be critical for improving outcomes in future pandemics.

## 5. Conclusions

The breastfeeding rate declined during and after the COVID-19 pandemic. This rate was affected not only by the pandemic but also by changes in health seeking behavior since the pandemic period.

As the world becomes increasingly interconnected, future pandemics are considered inevitable, and it is essential that we strengthen our preparedness based on the lessons learned from COVID-19. Moreover, our findings indicate that the effects of the pandemic on breastfeeding practices and maternal experiences are still evident, underscoring the need for continued efforts to address these ongoing challenges.

## Figures and Tables

**Table 1:** Participant characteristics.

	Before Pandemic (*n* = 812)	During Pandemic (*n* = 1602)	After Pandemic (*n* = 214)	*p*-Value
Gestational weeks	39.3 ± 1.1	39.4 ± 1.1	39.5 ± 1.0	0.29
Sex (boy (%))	428 (52.7%)	806 (50.3%)	103 (48.1%)	0.38
Delivery method (normal delivery (%))	664 (81.8%)	1262 (78.8%)	179 (83.6%)	0.09
Birth weight (g)	3049.1 ± 363.4	3078.9 ± 372.5	3067.1 ± 352.0	0.44
Body length (cm)	48.7± 1.8	48.7 ± 1.9	48.8 ± 1.8	0.54

Continuous variables were analyzed using analysis of variance, whereas categorical variables were analyzed using the chi-square test.

**Table 2:** Rates of exclusive breastfeeding, mixed feeding, and formula feeding alone. The rates of exclusive breastfeeding were compared among the groups (chi-square test).

	Before Pandemic (*n* = 812)	During Pandemic (*n* = 1602)	After Pandemic (*n* = 214)	*p*-Value
Feeding details at one month	343 (42.2%)/	450 (28.1%)/	49 (22.9%)/	<0.001
mother’s milk only/	395 (48.7%)/	959 (59.9%)/	142 (66.4%)/	
mother’s milk and formula milk/formula milk	74 (9.1%)	193 (12.1%)	23 (10.8%)	
Exclusive breastfeeding (%)	343 (42.2%)	450 (28.1%)	49 (22.9%)	<0.001
Any breastfeeding (%)	738 (90.9%)	1409 (88.0%)	191 (89.3%)	0.09

Feeding details at one month is valuable name and mother milk only/mother milk and formula milk/formula milk are the details of them. The numbers show their rate respectively.

**Table 3:** Results of logistic regression on exclusive breastfeeding. Periods before, during and after pandemic, maternal age, and delivery mode were analyzed.

Variables	Odds Ratio	95% C.I.	*p*-Value
Before pandemic (reference)			
During pandemic	0.54	0.45–0.64	<0.001
After pandemic	0.41	0.29–0.57	<0.001
Maternal age (year)	0.98	0.97–0.99	<0.05
Delivery mode (cesarian section)	0.80	0.64–0.99	<0.05

## Data Availability

The data presented in this study are available on request from the corresponding author. The data are not publicly available due to privacy and ethical reasons.

## Abbreviations

The following abbreviations are used in this manuscript:

COVID-19	Coronavirus disease 2019

## References

[1] Rollins N.C., Bhandari N., Hajeebhoy N., Horton S., Lutter C.K., Martines J.C., Piwoz E.G., Richter L.M., Victora C.G. (2016). Why invest, and what it will take to improve breastfeeding practices?. Lancet.

[2] Purkiewicz A., Regin K.J., Mumtaz W., Pietrzak-Fiećko R. (2025). Breastfeeding: The Multifaceted Impact on Child Development and Maternal Well-Being. Nutrients.

[3] Wallenborn J.T., Levine G.A., Carreira Dos Santos A., Grisi S., Brentani A., Fink G. (2021). Breastfeeding, Physical Growth, and Cognitive Development. Pediatrics.

[4] Patnode C.D., Henrikson N.B., Webber E.M., Blasi P.R., Senger C.A., Guirguis-Blake J.M. (2025). Breastfeeding and Health Outcomes for Infants and Children: A Systematic Review. Pediatrics.

[5] Luo C., Chen W., Cai J., He Y. (2024). The mechanisms of milder clinical symptoms of COVID-19 in children compared to adults. Ital. J. Pediatr..

[6] Shekerdemian L.S., Mahmood N.R., Wolfe K.K., Riggs B.J., Ross C.E., McKiernan C.A., Heidemann S.M., Kleinman L.C., Sen A.I., Hall M.W. (2020). Characteristics and Outcomes of Children with Coronavirus Disease 2019 (COVID-19) Infection Admitted to US and Canadian Pediatric Intensive Care Units. JAMA Pediatr..

[7] Mulkey S.B., Bearer C.F., Molloy E.J. (2023). Indirect effects of the COVID-19 pandemic on children relate to the child’s age and experience. Pediatr. Res..

[8] Hwang T.J., Rabheru K., Peisah C., Reichman W., Ikeda M. (2020). Loneliness and social isolation during the COVID-19 pandemic. Int. Psychogeriatr..

[9] Magis-Weinberg L., Arreola Vargas M., Carrizales A., Trinh C.T., Muñoz Lopez D.E., Hussong A.M., Lansford J.E. (2025). The impact of COVID-19 on the peer relationships of adolescents around the world: A rapid systematic review. J. Res. Adolesc. Off. J. Soc. Res. Adolesc..

[10] Groß R., Conzelmann C., Müller J.A., Stenger S., Steinhart K., Kirchhoff F., Münch J. (2020). Detection of SARS-CoV-2 in human breastmilk. Lancet.

[11] Bhatt H. (2021). Should COVID-19 mother breastfeed her newborn child? A literature review on the safety of breastfeeding for pregnant women with COVID-19. Curr. Nutr. Rep..

[12] World Health Organization Coronavirus Disease (COVID-19): Breastfeeding. https://www.who.int/news-room/questions-and-answers/item/coronavirus-disease-covid-19-breastfeeding.

[13] Baker R.E., Mahmud A.S., Miller I.F., Rajeev M., Rasambainarivo F., Rice B.L., Takahashi S., Tatem A.J., Wagner C.E., Wang L.F. (2022). Infectious disease in an era of global change. Nat. Rev. Microbiol..

[14] Chertok I.A., Artzi-Medvedik R., Arendt M., Sacks E., Otelea M.R., Rodrigues C., Costa R., Linden K., Zaigham M., Elden H. (2022). Factors associated with exclusive breastfeeding at discharge during the COVID-19 pandemic in 17 WHO European Region countries. Int. Breastfeed. J..

[15] Ganho-Ávila A., Guiomar R., Sobral M., Pacheco F., Caparros-Gonzalez R.A., Diaz-Louzao C., Motrico E., Domínguez-Salas S., Mesquita A., Costa R. (2023). The impact of COVID-19 on breastfeeding rates: An international cross-sectional study. Midwifery.

[16] Victora C.G., Bahl R., Barros A.J., França G.V., Horton S., Krasevec J., Murch S., Sankar M.J., Walker N., Rollins N.C. (2016). Breastfeeding in the 21st century: Epidemiology, mechanisms, and lifelong effect. Lancet.

[17] Ruiz M.T., Oliveira K.F., Azevedo N.F., Paschoini M.C., Rodrigues W.F., Oliveira C.J.F., Oliveira J.F., Fonseca L.M.M., Wernet M. (2023). Breastfeeding prevalence in newborns of mothers with COVID-19: A systematic review. Rev. Bras. Enferm..

[18] Zain E., Watanabe Y., Fukui N., Hashijiri K., Motegi T., Ogawa M., Egawa J., Nishijima K., Someya T. (2025). Primiparity, older maternal age, COVID-19 pandemic, twin birth, and winter birth are associated with lower exclusive breastfeeding in Japanese mothers. Sci. Rep..

[19] Cinquetti M., Marchiotto C., Fingerle M., Salani M., Adami A., Dainese D., Magaraggia S., Rigotti E., Piacentini G. (2023). Breastfeeding rates fell in an Italian baby friendly hospital during the 2020 COVID-19 pandemic year and difficulties increased. Acta Paediatr. (Oslo Nor. 1992).

[20] Regner C.J., Zgierska A.E., Lennon R.P., Goldstein E. (2024). Enablers and Challenges of Breastfeeding During the COVID-19 Pandemic. WMJ Off. Publ. State Med. Soc. Wis..

[21] Jones H.E., Seaborne M.J., Mhereeg M.R., James M., Kennedy N.L., Bandyopadhyay A., Brophy S. (2023). Breastfeeding initiation and duration through the COVID-19 pandemic, a linked population-level routine data study: The Born in Wales Cohort 2018–2021. BMJ Paediatr. Open.

[22] Hamad R., Collin D.F., Gemmill A., Jackson K., Karasek D. (2023). The Pent-Up Demand for Breastfeeding Among US Women: Trends After COVID-19 Shelter-in-Place. Am. J. Public Health.

[23] Di Mario S., Gagliotti C., Cattaneo A., Battaglia S., Cisbani L., Franchi F. (2023). Impact of COVID-19 Pandemic on Breastfeeding by Family Vulnerability: An Observational Study Based on Record Linkage. Breastfeed. Med. Off. J. Acad. Breastfeed. Med..

[24] Lubbe W., Botha E., Niela-Vilen H., Reimers P. (2020). Breastfeeding during the COVID-19 pandemic-a literature review for clinical practice. Int. Breastfeed. J..

[25] Latorre G., Martinelli D., Guida P., Masi E., De Benedictis R., Maggio L. (2021). Impact of COVID-19 pandemic lockdown on exclusive breastfeeding in non-infected mothers. Int. Breastfeed. J..

[26] Brown A., Shenker N. (2021). Experiences of breastfeeding during COVID-19: Lessons for future practical and emotional support. Matern. Child Nutr..

[27] Ceulemans M., Verbakel J.Y., Van Calsteren K., Eerdekens A., Allegaert K., Foulon V. (2020). SARS-CoV-2 Infections and Impact of the COVID-19 Pandemic in Pregnancy and Breastfeeding: Results from an Observational Study in Primary Care in Belgium. Int. J. Environ. Res. Public Health.

[28] Dennis C.L., Hodnett E., Gallop R., Chalmers B. (2002). The effect of peer support on breast-feeding duration among primiparous women: A randomized controlled trial. CMAJ Can. Med. Assoc. J. J. l’Association Medicale Can..

[29] Gavine A., Shinwell S.C., Buchanan P., Farre A., Wade A., Lynn F., Marshall J., Cumming S.E., Dare S., McFadden A. (2022). Support for healthy breastfeeding mothers with healthy term babies. Cochrane Database Syst. Rev..

[30] Lyons G.C., Kay M.C., Duke N.N., Bian A., Schildcrout J.S., Perrin E.M., Rothman R.L., Yin H.S., Sanders L.M., Flower K.B. (2023). Social Support and Breastfeeding Outcomes Among a Racially and Ethnically Diverse Population. Am. J. Prev. Med..

[31] Chmielewska B., Barratt I., Townsend R., Kalafat E., van der Meulen J., Gurol-Urganci I., O’Brien P., Morris E., Draycott T., Thangaratinam S. (2021). Effects of the COVID-19 pandemic on maternal and perinatal outcomes: A systematic review and meta-analysis. Lancet. Glob. Health.

[32] Agrina A., Afandi D., Suyanto S., Erika E., Dewi Y.I., Helina S., Pramita D., Safira N. (2022). Analysis of Supporting Factors Associated with Exclusive Breastfeeding Practice in the Urban Setting during the COVID-19 Pandemic. Children.

[33] Pacheco F., Sobral M., Guiomar R., de la Torre-Luque A., Caparros-Gonzalez R.A., Ganho-Ávila A. (2021). Breastfeeding during COVID-19: A Narrative Review of the Psychological Impact on Mothers. Behav. Sci..

[34] Gao S., Su S., Zhang E., Liu R., Zhang Y., Wang C., Liu J., Xie S., Yin C., Yue W. (2022). Psychological health status in postpartum women during COVID-19 pandemic: A systematic review and meta-analysis. J. Affect. Disord..

[35] Patrick M., Venkatesh R.D., Stukus D.R. (2022). Social media and its impact on health care. Ann. Allergy Asthma Immunol. Off. Publ. Am. Coll. Allergy Asthma Immunol..

[36] Zhang X., Du L., Huang Y., Luo X., Wang F. (2024). COVID-19 information seeking and individuals’ protective behaviors: Examining the role of information sources and information content. BMC Public Health.

[37] Knutson B., Hsu T.W., Ko M., Tsai J.L. (2024). News source bias and sentiment on social media. PLoS ONE.

[38] Zahroh R.I., Cheong M., Hazfiarini A., Vazquez Corona M., Ekawati F.M., Emilia O., Homer C.S., Betrán A.P., Bohren M.A. (2024). The Portrayal of Cesarean Section on Instagram: Mixed Methods Social Media Analysis. JMIR Form. Res..

[39] Morse H., Brown A. (2022). The benefits, challenges and impacts of accessing social media group support for breastfeeding: A systematic review. Matern. Child Nutr..

[40] Van Lancker W., Parolin Z. (2020). COVID-19, school closures, and child poverty: A social crisis in the making. Lancet. Public Health.

[41] Rebouças P., Falcão I.R., Barreto M.L. (2022). Social inequalities and their impact on children’s health: A current and global perspective. J. Pediatr..

[42] Watakabe K., Kawada S., Horiuchi S., Asahiro R., Tanaka A., Tei K., Murano Y., Nakazawa T., Sakamaki K., Shoji H. (2026). Characteristics Associated with Infant Feeding with Both Breast Milk and Formula Milk. Nutrients.

[43] Kronborg H., Vaeth M. (2004). The influence of psychosocial factors on the duration of breastfeeding. Scand. J. Public Health.

